# Total Sealing Technique Using an Advanced Bipolar Vessel-Sealing System in Axillary Lymph Node Dissection: A Technical Note and Review of Clinical and Economic Outcomes

**DOI:** 10.3390/cancers18061016

**Published:** 2026-03-20

**Authors:** Naoya Ikeda, Takuya Nagata, Teiji Umemura, Haruhito Kinoshita, Shinichiro Kashiwagi

**Affiliations:** 1 Department of Surgery, KIWA Hospital, Wakayama 648-0085, Japan; 2 Department of Forensic Medicine, Wakayama Medical University, Wakayama 641-8509, Japan; 3 Department of Surgery, Toho University Ohashi Medical Center, Tokyo 153-8515, Japan; 4 Department of Breast Surgical Oncology, Graduate School of Medicine, Osaka Metropolitan University, 1-4-3 Asahi-machi, Abeno-ku, Osaka 545-8585, Japan

**Keywords:** breast cancer, axillary lymph node dissection, total sealing technique, advanced bipolar vessel-sealing system, LigaSure™ Exact, advanced energy device, Harmonic scalpel, lymphedema, seroma, hospital stay, health economics

## Abstract

**Background**: Axillary lymph node dissection (ALND) remains necessary for selected patients with breast cancer but is associated with lymphatic morbidity, including seroma formation and breast cancer-related lymphedema (BCRL). The Total Sealing Technique (TST) is a technique-centered operative concept that emphasizes systematic sealing of lymphatic and vascular structures during ALND. **Methods**: This review integrates mechanistic rationale and clinical evidence derived from comparative cohort studies evaluating TST (using advanced bipolar vessel-sealing systems) versus conventional electrocautery (CONV). Key perioperative and long-term outcomes are summarized quantitatively. **Results**: In a comparative cohort of total mastectomy with ALND, TST significantly reduced total drainage volume (360.5 ± 187.9 vs. 820.6 ± 661.6 mL; *p* < 0.001) and shortened time to drain removal (4.8 ± 1.3 vs. 6.8 ± 2.1 days; *p* < 0.001). Postoperative hospital stay was reduced by 3.7 days on average (5.9 ± 1.3 vs. 9.6 ± 3.4 days; *p* < 0.001). The incidence of seroma decreased from 65.9% to 28.6% (*p* = 0.001), with fewer aspiration procedures (1.8 vs. 4.6 per patient; *p* = 0.022). Importantly, long-term follow-up demonstrated a statistically significant reduction in BCRL incidence (2.9% vs. 22.2%; *p* = 0.028). Operative time and blood loss were not increased. **Conclusions**: Current single-center data indicate that TST is associated with substantial reductions in postoperative lymphatic morbidity and a statistically significant decrease in BCRL incidence. While independent multicenter validation is warranted, TST represents a reproducible technique-centered approach with meaningful clinical impact in ALND.

## Simple Summary

Axillary lymph node dissection (ALND) remains necessary for selected patients with breast cancer, but it is frequently associated with lymphatic complications such as seroma formation and breast cancer-related lymphedema (BCRL), which can significantly impair long-term quality of life. The Total Sealing Technique (TST) is a technique-centered surgical approach that emphasizes systematic sealing of lymphatic and vascular structures during axillary dissection. Clinical cohort data demonstrate that TST reduces postoperative drainage, shortens drain placement and hospital stay, lowers seroma burden, and is associated with a statistically significant decrease in BCRL incidence compared with conventional electrocautery-based ALND. These findings suggest that optimization of surgical technique—rather than device substitution alone—may meaningfully influence long-term lymphatic outcomes. While further independent validation is warranted, TST represents a reproducible operative strategy aimed at minimizing morbidity when ALND remains indicated.

## 1. Introduction

Breast cancer is the most commonly diagnosed cancer in women worldwide and remains a major contributor to cancer-related morbidity and mortality, with an estimated 2.3 million new cases and 670,000 deaths in 2022 [1]. Surgical treatment is fundamental to curative management, providing local disease control and accurate pathological staging to guide adjuvant therapy. Despite its oncologic role, axillary lymph node dissection (ALND) is associated with substantial short- and long-term morbidity [2,3]. While recent clinical trials have increasingly supported de-escalation of axillary surgery in order to reduce morbidity—particularly in the neoadjuvant setting, where ALND is increasingly avoided in selected responders [4]—ALND remains an essential procedure for patients with persistent nodal disease or extensive axillary involvement. In these cases, efforts to minimize surgical trauma and prevent lymphatic complications remain of paramount importance. Lymphorrhea and seroma formation are frequent early complications, often leading to prolonged drainage, delayed recovery, and repeated outpatient interventions. More importantly, breast cancer-related lymphedema (BCRL) represents a chronic and potentially lifelong condition that adversely affects physical function, psychosocial well-being, and overall quality of life while generating sustained healthcare costs [2,5]. Prospective cohort data demonstrate a clear association between the extent of axillary intervention and cumulative BCRL incidence, with five-year rates exceeding 25–30% following ALND combined with regional nodal irradiation [3]. Thus, even within an era of surgical de-escalation, morbidity reduction in patients who require ALND remains a clinically relevant priority. From both biological and technical perspectives, the quality of lymphatic control during ALND is central to postoperative outcomes. Conventional ALND is typically performed using monopolar electrocautery, which effectively achieves hemostasis but may incompletely seal lymphatic channels and cause collateral thermal injury [6,7]. Experimental and clinical observations suggest that insufficient lymphatic sealing may contribute to persistent leakage, seroma formation, and chronic inflammatory changes that predispose to BCRL. Energy-based vessel-sealing systems, including bipolar platforms such as LigaSure™, have been associated with reduced drainage volume, earlier drain removal, and shorter hospital stay in several comparative studies [6,8,9]. While these advanced energy devices (i) can generally reduce operative time and early postoperative drainage volume, their long-term impact on the incidence of BCRL remains inconsistent across the literature. This suggests that the use of a specific device alone (ii) may not be sufficient to ensure superior lymphatic outcomes, and that the underlying surgical strategy plays a critical role. Consequently, the Total Sealing Technique (TST) was developed as a standardized, technique-centered approach (iii) to ALND, emphasizing systematic sealing of dissected tissues prior to transection in order to enhance lymphatic control. Unlike selective vessel coagulation, TST aims to comprehensively close microlymphatic channels within axillary adipose tissue. Clinical cohort studies have suggested reductions in postoperative lymphatic morbidity and a lower incidence of BCRL following TST-based ALND [6,10]. The present review synthesizes current evidence on ALND techniques, clearly distinguishing between energy devices in general, specific vessel-sealing platforms, and TST as an operative concept, and discusses their potential clinical and health economic implications.

## 2. Pathophysiology of Postoperative Morbidity After ALND

Axillary lymph node dissection disrupts a complex network of lymphatic channels, venous tributaries, nerves, and adipose tissue. Postoperative morbidity reflects the combined effects of mechanical disruption, thermal injury, inflammation, and tissue remodeling [2,5,7]. Understanding these mechanisms is essential when evaluating whether refinement of surgical technique can influence both short- and long-term outcomes.

### 2.1. Lymphorrhea and Seroma Formation

Lymphorrhea results from persistent leakage of transected lymphatic vessels after ALND. Unlike blood vessels, lymphatic channels have thin walls and lack intrinsic clotting mechanisms, rendering them vulnerable to postoperative leakage [6,11]. The durability of intraoperative lymphatic sealing therefore plays a central role in postoperative drainage. Monopolar electrocautery achieves hemostasis through thermal coagulation but may provide variable lymphatic sealing. Experimental models have demonstrated lower burst pressures and higher seal failure rates with monopolar instruments compared with advanced bipolar vessel-sealing systems or ultrasonic systems [12]. These findings suggest that differences in sealing strength may contribute to postoperative lymphatic leakage. Seroma formation represents the clinical expression of persistent lymphorrhea combined with inflammatory exudation. Risk factors include the extent of dissection, surgical technique, energy modality, and patient-related variables such as obesity [13,14]. Clinically significant seroma may require repeated aspirations and is associated with increased infection risk and potential delays in adjuvant therapy [6].

### 2.2. Inflammatory Response and Thermal Injury

The incidence of seroma has also been shown to vary according to surgeon-related factors, underscoring the role of operative technique [15]. A key contributor is thermal injury associated with surgical energy devices. Monopolar electrocautery generates lateral thermal spread beyond the immediate dissection plane, resulting in collateral tissue injury [7,16]. Thermal injury induces adipocyte necrosis, microvascular thrombosis, and release of pro-inflammatory cytokines, thereby amplifying capillary permeability and interstitial fluid exudation [17,18]. Persistent inflammation and subsequent fibrosis within the axilla may further compromise collateral lymphatic pathways and reduce compensatory drainage capacity, exacerbating lymphatic dysfunction [5].

### 2.3. Breast Cancer-Related Lymphedema (BCRL)

Breast cancer-related lymphedema is a progressive and often irreversible condition characterized by chronic limb swelling, fibrosis, and adipose deposition. Its pathogenesis is multifactorial, involving reduced lymphatic transport capacity, increased lymphatic load, and impaired lymphangiogenesis [5]. ALND remains one of the strongest independent risk factors for BCRL, particularly when combined with regional nodal irradiation [3]. Importantly, accumulating evidence suggests that the incidence of BCRL is strongly influenced by surgical technique and surgeon-dependent factors. From a biological perspective, the extent and quality of lymphatic injury incurred during ALND determine the residual functional lymphatic reserve within the axilla. Use of monopolar electrocautery may represent a major modifiable risk factor for BCRL, as its lateral thermal spread can damage perilymphatic tissues beyond the intended dissection plane. Heat-induced injury promotes chronic inflammation, fibrosis, and obliteration of microlymphatic channels, thereby impairing compensatory lymphatic drainage and predisposing patients to progressive lymphatic failure. Conversely, surgical strategies that minimize collateral thermal injury while achieving comprehensive lymphatic sealing may better preserve microlymphatic continuity and lymphatic reserve. In this context, technique-centered approaches emphasizing precise dissection and controlled energy delivery offer a biologically plausible means of reducing long-term BCRL risk [10,19].

## 3. Conventional Electrocautery Versus Energy-Based Devices

### 3.1. Conventional Electrocautery in Axillary Surgery

Monopolar electrocautery has long been the standard technique for tissue dissection and hemostasis in modified radical mastectomy because of its simplicity, wide availability, and effectiveness in reducing intraoperative blood loss [20,21]. In axillary lymph node dissection (ALND), monopolar cautery allows rapid division of soft tissue and reliable coagulation of visible vessels. However, the biological effects of electrocautery extend beyond simple vessel coagulation. Temperatures exceeding 200 °C may induce tissue carbonization, fat necrosis, and collateral thermal injury to adjacent lymphatic and neural structures [7]. Importantly, the axillary region contains not only identifiable blood vessels but also an extensive and diffuse network of microlymphatic channels embedded within adipose and connective tissues. Thermal coagulation may seal these channels transiently; however, experimental studies have demonstrated that lymphatic channels closed by cauterization can reopen in the postoperative period, resulting in persistent lymphorrhea. Clinically, this may manifest as increased drain output, prolonged drain duration, and a higher incidence of seroma formation following ALND [10,11]. Bipolar electrocautery represents a refinement of conventional thermal technology and allows more precise energy delivery compared with monopolar devices. While bipolar forceps are suitable for targeted hemostasis, they remain fundamentally designed for focal vessel coagulation rather than en bloc sealing of the entire lymphatic-bearing tissue plane. In the context of axillary dissection—where numerous microscopic lymphatic channels are diffusely distributed—achieving comprehensive and durable lymphatic closure using point-by-point coagulation may be technically demanding and potentially incomplete. Similarly, surgical clips provide reliable mechanical ligation of discrete vessels and are cost-effective. Nevertheless, their pinpoint application makes them less practical for addressing the dense and widespread lymphatic network of the axilla. Complete application to all dissected lymphatic tissues would require the placement of numerous clips, which may prolong operative time and could interfere with postoperative imaging assessment. Taken together, although conventional electrocautery techniques remain effective for hemostasis and are widely accepted in axillary surgery, their ability to achieve consistent, durable sealing of the extensive lymphatic network is limited. These limitations provide the rationale for exploring advanced bipolar vessel-sealing systems such as the LigaSure™ Exact, which are designed to deliver controlled energy with uniform tissue compression, potentially enabling more reliable sealing of both vascular and lymphatic structures during axillary dissection.

### 3.2. Limitations of Conventional Techniques

Postoperative outcomes after conventional ALND show considerable variability. Reported seroma rates range widely, from 15% to over 60% [13,18], reflecting not only patient-related factors but also surgeon-dependent differences in technique [15]. Variability in lymphatic control—including extent of cautery use and tissue handling—may contribute to inconsistent outcomes across institutions. These observations highlight the importance of operative technique in determining postoperative morbidity [22].

### 3.3. Advanced Bipolar Vessel-Sealing Systems (LigaSure*™* Exact)

From a clinical standpoint, advanced bipolar vessel-sealing systems such as LigaSure™ provide a reproducible method for controlling lymphatic and vascular structures during axillary dissection. By enabling consistent sealing across tissue bundles, these devices have been associated with reductions in postoperative drainage volume, earlier drain removal, and shorter hospital stay—outcomes that directly influence perioperative recovery and healthcare resource utilization [6,8,9]. Clinical trials and meta-analyses have consistently demonstrated that LigaSure™ reduces postoperative drain volume and duration compared with conventional techniques. The meta-analysis by Imran et al., which synthesized data from randomized and observational studies, confirmed significant reductions in drainage volume, drain duration, and length of hospital stay without increases in operative time or complication rates [8,9,23]. Nevertheless, most studies evaluating LigaSure™ have treated the device primarily as a substitute for electrocautery rather than as part of a comprehensive surgical strategy. Surgeons often employ hybrid approaches, combining advanced bipolar sealing for selected vessels with electrocautery for general dissection, which may dilute the potential benefits of complete lymphatic sealing [6,10].

### 3.4. Ultrasonic Devices (Harmonic*^®^*)

Ultrasonic devices such as Harmonic^®^ utilize high-frequency vibration to cut and coagulate tissue at lower temperatures than electrocautery, thereby reducing lateral thermal spread [24,25]. Randomized trials and meta-analyses have demonstrated reductions in postoperative drainage volume, seroma formation, and intraoperative blood loss compared with electrocautery [26,27]. Modest reductions in length of hospital stay have also been reported in some series. Similar to advanced bipolar systems, ultrasonic devices have generally been studied as tools rather than as components of a standardized dissection strategy. Long-term endpoints, including BCRL incidence, are infrequently reported, limiting conclusions regarding sustained benefit [5,26].

### 3.5. Lessons from Advanced Energy Device Studies

Collectively, studies of advanced bipolar vessel-sealing systems and ultrasonic systems demonstrate that improved control of lymphatic and vascular structures can reduce early postoperative morbidity after ALND [6,23,28]. At the same time, variability in technique and workflow across studies suggests that technological capability alone may not fully determine outcomes. These findings support further exploration of structured, technique-centered approaches to lymphatic control during axillary surgery [6,10]. To facilitate a clearer understanding of these technological differences, a structured comparison of surgical modalities, energy mechanisms, clinical advantages, and limitations is summarized in Table 1.

## 4. Total Sealing Technique (TST): Concept, Technique, and Rationale

### 4.1. Conceptual Framework and Surgical Procedure of TST

#### 4.1.1. Background and Rationale

The author’s prior experience in hepatobiliary and pancreatic surgery provided a unique perspective when expanding his clinical focus to breast surgery in 2017. A primary clinical observation during this transition was the remarkably high volume of postoperative drainage following conventional axillary lymph node dissection (ALND). This led to the hypothesis that conventional monopolar electrocautery fails to achieve permanent occlusion of small-caliber lymphatic channels, and that this incomplete sealing is the root cause of persistent lymphorrhea, prolonged drain placement, and seroma formation. To address this, the author reasoned that systematically sealing and transecting all dissected tissue bundles within the axilla—rather than relying on haphazard cauterization—would significantly reduce lymphatic leakage. A preliminary study was conducted to evaluate five different energy modalities: BiClamp^®^, SonoSurg^®^, THUNDERBEAT^®^, HARMONIC FOCUS^®^, and LigaSure™ Exact. Among these, the LigaSure™ Exact dissector demonstrated the most substantial reduction in drainage volume and offered superior maneuverability; consequently, it was selected for subsequent clinical implementation. Our comparative data confirmed that, relative to monopolar electrocautery, the Total Sealing Technique (TST) using LigaSure™ Exact significantly reduced total drainage volume, drain duration, length of hospital stay, and the incidence of seroma, while also facilitating an earlier initiation of adjuvant chemotherapy.

#### 4.1.2. Rationale for BCRL Reduction

The rationale for TST’s impact on reducing breast cancer-related lymphedema (BCRL) became evident during long-term patient follow-up. We observed a significantly lower incidence of BCRL in the TST group compared to the conventional group. Literature review suggests that the high thermal spread generated by monopolar electrocautery [7,29] causes collateral damage to preserved tissues, triggering chronic inflammatory responses and subsequent fibrosis of the remaining lymphatic vessels—a primary driver of BCRL. Furthermore, we concluded that persistent lymphorrhea and seroma formation themselves create a protein-rich environment that induces an inflammatory cascade and fibrotic scarring, further obstructing lymphatic flow [10]. By achieving meticulous occlusion of lymphatics while minimizing thermal spread to the surrounding microenvironment, TST effectively mitigates these two key pathophysiological factors, thereby preventing the onset of BCRL. The Total Sealing Technique (TST) was developed to address the inconsistent control of lymphatic channels during conventional axillary lymph node dissection (ALND). While traditional approaches primarily emphasize hemostasis, lymphatic sealing is a key determinant of postoperative lymphorrhea, seroma formation, and long-term breast cancer-related lymphedema (BCRL) [6]. TST is a technique-centered operative concept that prioritizes systematic sealing of all tissues designated for dissection before transection. Rather than selectively coagulating visible vessels, this approach aims to close both macroscopic lymphatic trunks and microscopic lymphatic channels embedded within axillary adipose tissue. By seeking comprehensive lymphatic control, TST is designed to interrupt the sequence from persistent leakage to inflammation, fibrosis, and long-term lymphatic dysfunction [10].

### 4.2. Surgical Technique and Standardization

TST is most commonly performed using an advanced bipolar vessel-sealing device. Dissection proceeds along defined anatomical planes using a consistent seal–divide–advance sequence, in which each tissue bundle is grasped, sealed, and divided in a uniform manner. Monopolar electrocautery is avoided to maintain consistency of lymphatic control throughout the axilla [10]. In principle, TST can be performed using alternative advanced bipolar sealing systems. However, smooth and rhythmical execution of the seal–divide–advance workflow depends substantially on the tip configuration and handling characteristics of the sealing device. In particular, the narrow and elongated jaw design of the LigaSure™ Exact dissector facilitates simultaneous grasping, sealing, and controlled division of axillary tissue bundles in a single-step manner. For this reason, while the conceptual framework of TST remains device-agnostic, the LigaSure™ Exact platform has proven to be particularly well suited to the consistent and reproducible implementation of the technique. This structured approach is intended to reduce surgeon-dependent variability and enhance reproducibility without requiring additional operative steps beyond familiarity with advanced bipolar sealing devices. The step-by-step operative workflow and key technical considerations for implementation of TST are summarized in Table 2.

### 4.3. Standardized Protocol and Technical Considerations

Building upon the standardized operative workflow described above, this section discusses critical technical considerations and potential pitfalls to ensure safe and reproducible implementation. While the learning curve for TST is relatively short for surgeons experienced in axillary anatomy, certain pitfalls must be avoided to ensure safety and efficacy. A primary concern is the risk of collateral thermal injury; the advanced bipolar sealing device should be activated at a distance of at least 2 mm from any identified nerves to maintain an adequate safety margin. Additionally, surgeons must ensure that the jaws of the device remain clean and free of carbonized debris throughout the procedure. In the case of the LigaSure™ Exact, the nano-coated jaws reduce tissue adhesion and carbonization, thereby minimizing this concern. However, with conventional sealing devices, accumulated tissue on the jaws can interfere with energy delivery, potentially resulting in incomplete sealing of lymphatic channels and subsequent lymphorrhea. Adherence to this standardized workflow ensures consistent and reproducible outcomes regardless of the surgical setting.

### 4.4. Safety, Contraindications, and Cautions

Regarding the clinical application of the Total Sealing Technique (TST), there are no absolute contraindications to the technique itself. However, specific caution is required in certain patient populations. In cases of extensive inflammatory breast cancer or bulky nodal involvement where normal tissue planes are significantly obliterated by tumor infiltration or peritumoral edema, the use of advanced bipolar devices must be approached with precision. In such scenarios, it is imperative to maintain safe thermal margins from major anatomical structures, specifically the long thoracic and thoracodorsal nerves, to prevent iatrogenic thermal injury. Furthermore, it must be emphasized that TST is an enhancement of, not a substitute for, sound oncological surgical principles. The use of sealing devices does not replace the fundamental requirement for meticulous anatomical identification of nerves and major vessels. Surgeons must ensure that the “Total Sealing” is applied only to the targeted lymphatic and vascular bundles after definitive identification and lateralization of the critical neurovascular structures.

### 4.5. Biological Rationale

Histopathological evaluation of lymphatic vessels treated with advanced bipolar vessel-sealing systems has demonstrated stable fusion of lymphatic vessel walls and durable luminal obliteration [6]. Advanced bipolar vessel-sealing induces collagen and elastin denaturation, forming a reinforced protein matrix at the sealed site. Microscopic analyses have shown preservation of surrounding tissue architecture with limited lateral thermal spread, findings consistent with controlled energy delivery [17]. These biological observations provide a mechanistic explanation for reduced postoperative lymphorrhea and seroma formation observed in clinical cohorts and support the plausibility of sustained lymphatic occlusion.

### 4.6. Clinical Outcomes Associated with TST

**Figure 1:**
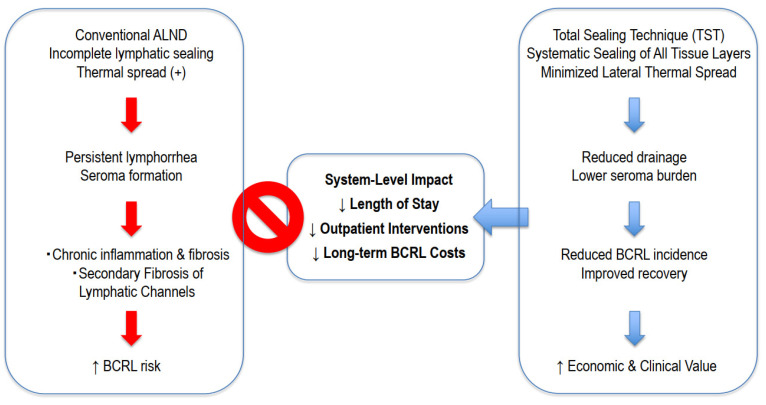
Conceptual Framework of Technique-Centered Lymphatic Preservation in ALND. The diagram illustrates the divergence in clinical and system-level outcomes between conventional methods and the Total Sealing Technique (TST). (**Left**) Conventional ALND: Incomplete lymphatic sealing combined with thermal spread leads to persistent lymphorrhea and seroma formation. This triggers chronic inflammation and secondary fibrosis of lymphatic channels, ultimately increasing the risk of breast cancer-related lymphedema (BCRL). (**Right**) Total Sealing Technique (TST): By employing systematic sealing of all tissue layers and minimizing lateral thermal spread, TST ensures reduced drainage and a lower seroma burden. This improved recovery pathway significantly reduces BCRL incidence. (**Center**) System-Level Impact: These clinical improvements translate into optimized healthcare resource utilization, characterized by reduced length of stay, fewer outpatient interventions, and lower long-term costs associated with BCRL management.

Comparative cohort studies have demonstrated that TST is associated with significantly lower total drainage volume, shorter drain duration, reduced seroma burden, and shorter postoperative hospitalization compared with conventional electrocautery-based ALND [6,10]. Importantly, long-term follow-up data demonstrated a statistically significant reduction in BCRL incidence from 22.2% to 2.9% (*p* = 0.028) following TST-based ALND [10]. Operative time, intraoperative blood loss, and overall perioperative complication rates were not increased in the reported series [6]. With regard to safety outcomes, we now explicitly state that in the published TST cohorts [6,10], there were no instances of major vascular injury or permanent nerve damage attributable to the use of the advanced bipolar vessel-sealing device. However, it should be acknowledged that these studies were not specifically powered to detect rare adverse events, and comprehensive safety evaluation—including standardized reporting of nerve injury, vascular trauma, and learning-curve effects—will require larger multicenter investigations. Available data derive primarily from single-center cohorts, and independent multicenter validation will be necessary to confirm generalizability and durability of these findings. Figure 1 illustrates the integrated mechanistic and system-level framework linking operative technique to lymphatic injury, downstream morbidity, and healthcare resource utilization, emphasizing the divergence between conventional axillary dissection and technique-centered lymphatic preservation.

## 5. Comparative Clinical Outcomes: TST Versus Energy-Based and Conventional Techniques

Clinical outcomes following axillary lymph node dissection (ALND) vary according to energy modality and operative workflow. A structured comparison of surgical techniques is presented in Table 1. Quantitative perioperative and long-term outcome differences between conventional and advanced bipolar vessel-sealing approaches are summarized in Table 3.

### 5.1. Length of Hospital Stay

Meta-analyses evaluating ultrasonic devices such as Harmonic^®^ report modest reductions in postoperative length of stay, typically ranging from approximately 1.0 to 1.5 days, compared with conventional electrocautery [26,28]. Similarly, pooled analyses of advanced bipolar vessel-sealing systems, including LigaSure™, demonstrate a mean reduction of approximately 1.5 days in hospital stay without increased operative time or complications [23]. In contrast, cohort data evaluating the Total Sealing Technique (TST) demonstrated a greater reduction in hospitalization, with a mean decrease of 3.7 days compared with conventional ALND (5.9 ± 1.3 vs. 9.6 ± 3.4 days; *p* < 0.001) [6]. While institutional discharge policies may influence absolute length of stay, the magnitude of reduction suggests that consistent lymphatic control may contribute to earlier readiness for discharge.

### 5.2. Seroma Formation and Lymphatic Output

Ultrasonic and advanced bipolar vessel-sealing devices consistently reduce postoperative drainage volume and drain duration relative to monopolar electrocautery [23,26]. However, reductions in seroma incidence have been heterogeneous across studies, and pooled analyses of LigaSure™ did not demonstrate a statistically significant reduction in seroma rates despite improvements in drainage parameters [23]. In contrast, TST cohort data demonstrate clear absolute reductions in clinically relevant seroma outcomes. Total drainage volume was reduced from 820.6 ± 661.6 mL to 360.5 ± 187.9 mL (*p* < 0.001), and drain duration decreased from 6.8 ± 2.1 to 4.8 ± 1.3 days (*p* < 0.001) [6]. Seroma incidence decreased from 65.9% to 28.6% (*p* = 0.001), and the mean number of aspiration procedures was reduced from 4.6 to 1.8 per patient (*p* = 0.022) [6]. These findings suggest that systematic sealing of lymphatic tissue bundles may influence not only early drainage parameters but also the downstream clinical burden of seroma.

### 5.3. Long-Term Outcomes and Breast Cancer-Related Lymphedema

Long-term outcomes such as breast cancer-related lymphedema (BCRL) are rarely reported in studies focused on device substitution [26,28]. Most randomized trials of ultrasonic or advanced bipolar devices emphasize short-term endpoints and lack extended follow-up, limiting conclusions regarding sustained lymphatic morbidity. In contrast, long-term follow-up data following TST-based ALND were associated with a statistically significant difference within a single-center cohort in BCRL incidence (22.2% with conventional electrocautery vs. 2.9% with TST; *p* = 0.028) [10]. Although these findings derive from single-center cohorts and require independent validation, they indicate that comprehensive lymphatic sealing at the time of initial dissection may influence long-term lymphatic reserve and clinical outcomes.

### 5.4. Biological Implications and the Immune Microenvironment

Recent advances in breast oncology have highlighted the critical role of the regional immune microenvironment in determining treatment outcomes. It is recommended to consider how surgical trauma and subsequent chronic inflammation—such as that caused by persistent seroma—may alter local immune signaling. Minimizing surgical morbidity through techniques like TST may have implications beyond physical recovery. Recent studies have explored the breast cancer immune microenvironment and the identification of predictive biomarkers for immunotherapy, emphasizing the importance of maintaining lymphatic and immunological integrity [30]. Chronic seroma and the associated inflammatory cytokine cascade may potentially interfere with the local immune landscape, possibly affecting the delivery or efficacy of systemic therapies. By achieving meticulous lymphatic sealing and reducing the inflammatory burden, TST may help preserve a more favorable regional microenvironment. Further research is warranted to investigate whether such surgical refinements can influence the expression of immune-related biomarkers or enhance the efficacy of emerging immunotherapeutic strategies in patients requiring ALND.

## 6. Health Economic Impact and Value Considerations

### 6.1. Framework for Economic Evaluation in ALND

Economic evaluation of surgical innovation should encompass costs across the continuum of care, including inpatient hospitalization, short-term outpatient management, and long-term chronic morbidity such as breast cancer-related lymphedema (BCRL) [31,32]. In ALND, downstream costs may exceed those of the index procedure when persistent complications occur [33,34]. It should be noted that the economic impact of technical modifications such as TST is highly dependent on regional healthcare systems and local discharge policies. The effect on length of hospital stay (LOS), in particular, varies substantially across healthcare environments. In countries with universal health insurance systems and a tradition of inpatient-centered postoperative recovery, such as Japan, patients are typically hospitalized until drain removal. In this setting, a reduction in postoperative drainage volume may directly translate into a shorter LOS, thereby generating measurable inpatient cost savings. Conversely, in healthcare systems where outpatient drain management is standard practice, the economic benefit of TST may be less apparent in terms of LOS reduction but may instead emerge through decreased outpatient visits for seroma aspiration and a lower incidence of long-term BCRL-related expenditures. The cost figures presented in this section are intended to be illustrative rather than definitive, providing a framework for understanding the potential economic impact of TST. Our analysis was primarily conducted from a hospital provider perspective, based on the Japanese healthcare reimbursement system using 2023 price years (converted at a rate of 1 USD ≈ 150 JPY). The key assumptions in this model include a standardized recovery pathway where LOS is directly influenced by drain duration, and the consistent use of advanced bipolar devices as per our protocol. We acknowledge that actual financial outcomes are subject to variability; sensitivity ranges suggest that the economic benefit is highly dependent on local purchase prices of disposables and regional discharge policies. Therefore, these values serve as a preliminary estimate to guide institutional decision-making across different healthcare landscapes. The major cost domains and illustrative economic implications across the continuum of care are summarized in Table 4.

### 6.2. Inpatient Costs: Length of Stay

Length of hospital stay (LOS) is a major determinant of inpatient expenditure after breast cancer surgery [35]. Published analyses estimate average per diem inpatient costs of approximately USD 2400–2500 following breast cancer surgery with ALND [2]. TST has been associated with a mean reduction in LOS of 3.7 days compared with conventional techniques [6]. Based on reported per diem estimates, this corresponds to an approximate inpatient cost difference of USD 8000–9000 per patient. Actual financial impact will vary according to healthcare system structure, reimbursement model, and discharge protocols; however, the magnitude of LOS reduction suggests potentially meaningful resource implications, particularly in high-volume centers [36,37].

### 6.3. Short-Term Outpatient Costs: Seroma Management

Seroma formation frequently necessitates repeated outpatient visits and aspiration procedures [38,39]. Published cost analyses estimate direct medical costs of approximately USD 200–500 per aspiration episode [40,41,42]. Patients requiring repeated aspirations may therefore incur cumulative outpatient costs of roughly USD 400–2000. Because TST significantly reduces both seroma incidence and the mean number of aspiration procedures (4.6 to 1.8 per patient) [6], a conservative estimate suggests potential short-term outpatient cost differences in the range of several hundred to approximately USD 1000 per patient. These figures do not include indirect costs such as productivity loss or caregiver burden.

### 6.4. Long-Term Costs: Prevention of BCRL

Breast cancer-related lymphedema (BCRL) is associated with sustained increases in healthcare utilization and expenditures [33,43]. Analyses have reported excess direct medical costs of approximately USD 14,000–15,000 per affected patient within the first two years following breast cancer treatment, with additional ongoing costs thereafter [33]. These expenditures reflect not only outpatient visits and rehabilitation but also complications such as recurrent cellulitis and hospital admissions. Given the observed reduction in BCRL incidence from 22.2% to 2.9% with TST [10], the absolute risk reduction of 19.3% translates into an expected short-term direct medical cost difference of approximately USD 2500–3000 per treated patient when calculated using published excess-cost estimates. Although these projections are context-dependent and require confirmation in prospective economic studies, they suggest that prevention of BCRL may offset a substantial portion of the initial device-related expenditure associated with advanced bipolar vessel-sealing systems (e.g., LigaSure™ Exact). Beyond early excess medical costs, prevention of BCRL through TST may also avert the need for costly long-term interventions. While conservative management—such as complex decongestive therapy and compression garments—remains the cornerstone of treatment, advanced microsurgical procedures including lymphaticovenular anastomosis (LVA) and vascularized lymph node transfer (VLNT) are increasingly performed in specialized centers for refractory cases [44]. These procedures entail substantial procedural costs, perioperative resource utilization, and long-term rehabilitative care. Therefore, reducing the incidence of BCRL from 22.2% to 2.9% [10] may meaningfully mitigate the cumulative lifetime economic burden associated with chronic lymphedema management. Importantly, the economic value of BCRL prevention must be interpreted within the context of diverse global healthcare systems. In countries operating under universal healthcare models—such as Japan and many European nations—hospital reimbursement is frequently linked to length of stay (LOS). In such settings, technologies that enhance reliable tissue sealing and reduce postoperative complications may provide direct economic relief by decreasing inpatient bed utilization. Conversely, in systems characterized by commercial insurance and outpatient-centered care (e.g., the United States), the economic advantage may be reflected more prominently in reduced outpatient visits for seroma aspiration, fewer procedure-related billing events, and diminished patient-level burdens associated with prolonged drain management. The cost–benefit profile may be even more consequential in low- and middle-income countries (LMICs). Although the upfront investment in advanced vessel-sealing devices and disposable instruments may represent a financial barrier, the long-term economic implications of preventing BCRL are potentially greater in these regions. In many LMICs, infrastructure for chronic lymphedema care—including specialized physiotherapy services and access to compression garments—is limited or unavailable. Under such conditions, preventing a lifelong and functionally debilitating condition through a standardized surgical technique may represent a highly cost-effective strategy. By averting chronic morbidity, TST may reduce indirect costs such as productivity loss and catastrophic out-of-pocket expenditures borne by patients and their families. Taken together, while institutional budget constraints may influence short-term adoption decisions, the prevention of BCRL through TST—implemented using contemporary vessel-sealing technology—has implications that extend beyond immediate postoperative savings, contributing to long-term economic sustainability across varied healthcare and socioeconomic environments.

### 6.5. Implications for Value-Based Surgical Care

Value-based healthcare emphasizes patient-centered outcomes relative to costs incurred [45]. In ALND, meaningful outcomes include functional recovery, reduction in chronic morbidity, and preservation of quality of life. By reducing short-term lymphatic complications and demonstrating a statistically significant decrease in BCRL incidence, TST may contribute to improved long-term value. Future multicenter studies incorporating standardized cost collection and formal cost-effectiveness analyses will be necessary to define the economic impact more precisely.

## 7. Limitations, Generalizability, and Future Directions

### 7.1. Limitations of the Current Evidence

Several limitations of the current evidence base should be acknowledged. A significant limitation is that available data on the Total Sealing Technique (TST) derive primarily from single-center cohort studies, which may be subject to center-specific effects—such as specialized surgical expertise, specific institutional protocols, or potential bias toward the technique. Although the observed reductions in lymphatic morbidity are substantial and demonstrate internal consistency, independent multicenter validation is essential to confirm the generalizability and durability of these findings across different surgical settings and expertise levels. Comparisons between TST and other energy-based approaches are further limited by heterogeneity in study design, patient selection, and outcome definitions. As emphasized in systematic reviews and meta-analyses of device-focused studies, most investigations prioritize short-term endpoints such as drainage volume and drain duration, whereas long-term outcomes—including breast cancer-related lymphedema (BCRL)—are rarely assessed in a standardized manner [28]. Given that BCRL is a delayed and progressive condition, the absence of extended follow-up in many device-comparison studies constrains meaningful evaluation of durable lymphatic preservation [2]. Furthermore, it is important to acknowledge that the development of BCRL is multifactorial, and surgical trauma is only one of several contributing elements. Significant confounders—including high body mass index (BMI), taxane-based chemotherapy, and regional nodal irradiation (RNI)—play critical roles in the final risk of BCRL [10]. While TST is designed to minimize the “surgical trigger” by reducing lymphatic leakage and chronic inflammation, these systemic and radiation-related factors remain decisive determinants of long-term lymphatic morbidity. Future studies should employ multivariate models to better isolate the independent effect of TST amidst these competing risk factors. Beyond methodological considerations, the broader applicability of TST must also be interpreted in light of economic and health system constraints. Routine implementation may face barriers in low- and middle-income countries (LMICs), where high initial capital investment and the cost of disposable advanced bipolar devices may limit accessibility.

Although projected long-term savings from reduced BCRL care are compelling, resource allocation in such settings often prioritizes basic surgical capacity and affordable oncologic services over high-cost energy platforms. This approach aligns with the Global Surgery 2030 framework, which underscores the need to strengthen essential surgical systems before adopting resource-intensive technologies [46]. Consequently, the adoption of TST for breast surgery may be deprioritized relative to other essential services or well-established laparoscopic procedures. The feasibility and scalability of TST should therefore be evaluated within the context of local institutional budgets, reimbursement structures, and the availability of shared equipment. Finally, because standardized reporting frameworks for lymphatic complications are inconsistently applied, adopting uniform definitions and patient-reported outcomes will be essential to accurately determine long-term clinical value.

### 7.2. Generalizability and Implementation

TST is conceptually adaptable across surgical settings because its defining principle—systematic sealing of lymphatic and vascular structures prior to transection—is not inherently restricted to a single proprietary device. In principle, other advanced bipolar vessel-sealing systems with comparable sealing reliability and thermal control could be used to implement the technique. However, effective and reproducible execution of TST depends on the ability to perform dissection, sealing, and division in a smooth, single-step sequence. Device characteristics—including jaw configuration, sealing length, and handling ergonomics—therefore play a meaningful role in practical implementation. In current clinical practice, the LigaSure™ Exact dissector has demonstrated particular suitability for consistent performance of TST owing to its narrow, elongated tip and controlled energy delivery profile. These features facilitate precise tissue handling within the confined axillary space and support uniform application of the seal–divide–advance workflow. Accordingly, while TST should be understood as a technique-centered concept rather than a device-specific innovation, optimal and reproducible implementation may depend on the use of a sealing platform with appropriate technical characteristics. Future comparative studies evaluating alternative advanced bipolar systems within a standardized TST framework would further clarify device-specific contributions.

### 7.3. Future Research Directions

The clinical management of the axilla is rapidly evolving toward de-escalation to minimize treatment-related morbidity. Recent large-scale evidence, such as the OPBC-05/EUBREAST-14R study [4], has demonstrated that omitting ALND in patients who achieve a clinical node-negative status (ycN0) after neoadjuvant chemotherapy—following targeted axillary dissection (TAD) or sentinel lymph node biopsy—is increasingly feasible and supported by oncologic safety data. However, despite this trend, ALND remains an oncological necessity for a subset of patients, including those with persistent nodal disease after neoadjuvant therapy or those with extensive initial axillary involvement where nodal conversion is not achieved. In these scenarios, where “mandatory ALND” is performed, the risk of BCRL remains a formidable challenge. The Total Sealing Technique (TST) provides a critical “secondary prevention” strategy in this specific clinical context. By optimizing the surgical execution of a high-risk procedure that cannot yet be avoided, TST ensures that even patients requiring radical axillary surgery can benefit from a minimized inflammatory burden and preserved lymphatic integrity. Future research should prioritize prospective multicenter studies comparing TST with contemporary ALND approaches across diverse healthcare systems. Standardized long-term follow-up—particularly with respect to BCRL incidence and severity—will be critical to validate the durability of benefit. Beyond technical refinements such as TST, several emerging strategies aim to preserve or reconstruct the lymphatic system during ALND. Since 2007, Axillary Reverse Mapping (ARM) has been developed to distinguish the lymphatic drainage pattern of the upper extremity from that of the breast [47,48]. A large prospective study of ARM reported that the incidence of lymphedema at 26 months was 6.5% in patients who underwent ALND [49]. In comparison, our clinical data showed that performing ALND with TST resulted in a 2.9% BCRL incidence, suggesting that this standardized sealing approach may offer a technically simpler alternative, although direct comparative trials are lacking. More recently, Immediate Lymphatic Reconstruction (ILR), also known as the Lymphatic Microsurgical Preventing Healing Approach (LYMPHA), has gained increasing attention [50]. This involves performing immediate lymphaticovenular anastomosis (LVA) at the time of ALND to restore lymphatic continuity. However, performing these complex microsurgical procedures in daily practice remains challenging and requires further high-level evidence [50]. Furthermore, ILR entails significantly higher healthcare costs, including the need for specialized microsurgical equipment, increased operative time, and the involvement of highly trained reconstructive teams. Consequently, there is an urgent need for scalable, reproducible, and cost-effective procedures that provide consistent results across various surgical settings. While ARM and ILR represent biologically restorative but technically and economically demanding strategies, TST provides a practical, technique-centered approach focused on the systematic closure of lymphatic vessels. This is achieved by ensuring effective sealing while minimizing thermal-induced lymphangitis in preserved lymphatics. Future studies are warranted to determine whether TST can be combined with these biological strategies to confer synergistic benefits.

## 8. Educational and Training Implications

### 8.1. Facilitating Consistency in Axillary Surgery

Inter-surgeon variability remains a factor in postoperative outcomes following axillary lymph node dissection (ALND). Differences in dissection technique and tissue handling can contribute to heterogeneity in lymphatic complications. While advanced energy devices do not replace the necessity for anatomical expertise or surgical judgment, the Total Sealing Technique (TST) provides a structured framework that may help minimize technical variability. Once anatomical landmarks are identified, the systematic seal–divide–advance sequence offers a reproducible method for tissue sealing. By following this operative algorithm, surgeons can achieve a consistent quality of lymphatic ligation along the dissection plane. Rather than standardizing the surgery itself, TST serves as a standardized technical adjunct that supports uniform tissue management across different levels of surgical experience.

### 8.2. Implications for Surgical Education

The integration of TST principles into surgical training may support the development of reliable lymphatic control strategies. Importantly, TST-based training is not a substitute for core competencies; rather, it reinforces them—including the three-dimensional understanding of axillary anatomy and the deliberate identification of tissue planes. Educational emphasis on relaxed tissue positioning, avoidance of excessive traction, and awareness of collateral thermal spread promotes the safe and controlled use of energy. These principles align with broader surgical training objectives and remain applicable regardless of the specific device platform used. Structured teaching approaches, such as video-based instruction and supervised implementation, focus on refining intraoperative judgment and technique execution. By prioritizing these foundational skills alongside the use of advanced energy devices, TST-oriented education may contribute to more predictable procedural outcomes and durable skill acquisition. Accordingly, the step-by-step surgical procedure and essential technical tips for axillary lymph node dissection using TST are summarized in Table 2.

## 9. Global and Health Policy Perspectives

Patterns of breast cancer surgery and perioperative care vary internationally according to healthcare systems and reimbursement models [51,52,53]. While advanced microsurgical strategies like ILR are gaining attention, their implementation is often hindered by global disparities in access to specialized expertise and expensive infrastructure [34,46]. In this context, TST offers a scalable alternative. Unlike ILR, which requires microsurgical equipment and extended operative time, TST leverages existing surgical workflows. Although the LigaSure™ Exact dissector incurs an additional disposable cost (approximately USD 600–700), this must be weighed against potential savings from shorter hospitalizations, fewer seroma-related interventions, and reduced BCRL management [6,10,54]. From a health policy perspective, such technique-centered innovations that reduce chronic morbidity without requiring specialized personnel may contribute to more sustainable cancer care across diverse economic settings.

## 10. Conclusions

Axillary lymph node dissection (ALND) remains an oncological necessity for selected patients with breast cancer; however, its long-term clinical value increasingly depends on minimizing treatment-related morbidity. The Total Sealing Technique (TST) represents a technique-centered reframing of axillary surgery by placing systematic lymphatic control at the center of the operative strategy. Available clinical data demonstrate meaningful reductions in postoperative lymphorrhea, seroma formation, and length of hospital stay, coupled with a statistically significant decrease in breast cancer-related lymphedema (BCRL). Beyond short-term perioperative improvements, the observed reduction in BCRL incidence (from 22.2% to 2.9%) suggests that meticulous intraoperative lymphatic sealing may positively influence long-term lymphatic reserve and survivorship outcomes. Furthermore, illustrative economic estimates indicate that a 3.7-day reduction in hospitalization could correspond to an approximate inpatient cost difference of USD 8000–9000 per patient, complemented by additional short-term outpatient savings from reduced seroma interventions and projected reductions in BCRL-associated expenditures. While these figures are context-dependent and require confirmation through formal economic analyses, they underscore the potential system-level implications of technique-driven morbidity prevention. As breast cancer care increasingly emphasizes survivorship, quality of life, and the sustainability of healthcare delivery, surgical strategies that prevent chronic morbidity at the time of initial intervention assume growing importance. TST represents a reproducible, scalable, and biologically rational approach that integrates technical precision with long-term outcome optimization. While independent multicenter validation and standardized economic evaluation are warranted to further define its role, current evidence supports further investigation of comprehensive lymphatic sealing as a potentially important principle in modern axillary surgery.

## Figures and Tables

**Table 1:** Comparison of Surgical Techniques and Energy-Based Devices in ALND *.

Technique	Energy Modality	Mechanism of Action	Study Type	Clinical Advantages	Limitations/Concerns
Conventional ALND	Monopolar electrocautery	High-frequency current via active electrode	Observational cohorts	Low cost and widely available	There is a high risk of thermal damage to surrounding tissues due to high lateral thermal spread.
Bipolar electrocautery-based ALND	Bipolar forceps	Current between two poles	Retrospective/Observational studies	Precise application; lower thermal spread than monopolar	The limited amount of tissue that can be treated at once makes it challenging to cover the entire dissection range.
LigaSure™-assisted ALND	Bipolar vessel-sealing	Radiofrequency energy with integrated pressure feedback	RCTs/meta-analyses	Reliable sealing of vessels up to 7 mm; reduced blood loss. No ligation required	Higher initial equipment cost; potential for technological dependency
Harmonic^®^-assisted ALND	Ultrasonic energy	Ultrasonic vibration	RCTs/meta-analyses	Enabling speedy procedures through simultaneous cutting and coagulation with minimal thermal injury.	Higher initial equipment cost; Lower burst pressure for large vessels compared to advanced bipolar vessel-sealing systems, along with extremely high jaw temperatures, necessitates cautious handling.
Total Sealing Technique (TST) using LigaSure™ Exact	Bipolar vessel-sealing (systematic sealing)	Radiofrequency energy with integrated pressure feedback	Single-center studies	Systematic sealing of all dissected tissue layers, including lymphatic and vascular structures	Higher initial equipment cost; potential for technological dependency. It takes some skill to master.

* ALND: Axillary Lymph Node Dissection.

**Table 2:** Standardized Operative Workflow and Key Technical Points for the Total Sealing Technique (TST) Using the LigaSure™ Exact.

**1. Entry into the Axillary Cavity** Make an incision in the deep pectoral fascia along the lateral border of the pectoralis minor muscle to gain access to the axillary cavity. Using a LigaSure™ Exact dissector (LGSED), perform tissue dissection with a seal-and-divide technique. ⋅ Technical Tip: Maintain a sufficiently thin dissection plane so that the dorsal jaw of the LGSED remains visible during activation.
**2. Identification of the Axillary Vein** Identify the axillary vein. As dissection proceeds along the lateral border of the pectoralis minor muscle, the medial pectoral nerve and medial pectoral vein are exposed. ⋅ Technical Tip: Tracing the medial pectoral vein proximally facilitates identification of the axillary vein.
**3. Dissection Along the Inferior Border of the Axillary Vein and Preservation of Arm Lymphatic Channels** Dissection is performed along the inferior border of the axillary vein. Numerous lymphatic channels draining from the upper limb are present in this region. ü Technical Tip: To preserve the arm lymphatic channels, avoid complete skeletonization of the axillary vein. Maintain the dissection plane slightly caudal to the vein.
**4. Management of the Lateral Thoracic Vein** After identification of the thoracodorsal artery, vein, and nerve, the lateral thoracic vein is doubly sealed using an LGSED and subsequently divided. Routine ligation is not required. ⋅ Technical Tip: Sealing should be performed under minimal tension. Excessive traction or overextension of the vessel may result in incomplete sealing.
**5. Dissection of the Axillary Fat Pad from the Thoracodorsal Bundle** The axillary fibrofatty tissue containing lymph nodes (axillary fat pad) is retracted caudally and completely dissected from the thoracodorsal bundle (thoracodorsal artery, vein, and nerve) using the TST. ⋅ Technical Tip: Dissection should be performed in a cranial-to-caudal direction. Approaching from the caudal side may increase the risk of peripheral injury.
**6. Dissection of the Axillary Fat Pad from the Long Thoracic Nerve** The axillary fat pad is retracted laterally and carefully dissected from the long thoracic nerve. Using the TST, the fat pad is dissected in a caudal-to-cranial direction toward the inferior border of the axillary vein, maintaining a margin of at least 2 mm from the long thoracic nerve. Completion of this step marks the end of the medial portion of the Level I dissection. ⋅ Technical Tip (Prevention of Long Thoracic Nerve Injury): Retract the en bloc axillary fat pad laterally to facilitate clear identification of the long thoracic nerve before dissection. ⋅ Technical Tip (Avoidance of Thermal Injury): When performing the TST with the LGSED, maintain a distance of at least 2 mm from the nerve to minimize the risk of thermal damage.
**7. Completion of Level I Lymph Node Dissection** Level I axillary lymph node dissection is completed by en bloc removal of all fat pad within the region defined by the following four anatomical boundaries: ⋅ Medial boundary: Lateral border of the pectoralis minor muscle. ⋅ Lateral boundary: Anterior border of the latissimus dorsi muscle. ⋅ Superior boundary: Inferior border of the axillary vein. ⋅ Inferior boundary: Level of the fourth to fifth intercostal space, corresponding to the course of the lateral thoracic vessels.

**Table 3:** Comparative Clinical Outcomes of Advanced Bipolar Vessel-Sealing Systems vs. Conventional ALND.

Outcome Parameter	Conventional Method	Advanced Bipolar Vessel-Sealing Systems (e.g., LigaSure™)	Key Findings/Rationale
Postoperative Drainage	Baseline output	Significantly Reduced	Effective sealing of lymphatic leakage
Drain Duration	Standard (e.g., >7 days)	Shorter Duration	Effective sealing of lymphatic leakage
Incidence of Seroma	15–30%	Markedly Lower	Effective sealing of lymphatic leakage
Incidence of * BCRL	22.2%	2.9%	Prevention of thermal damage to surrounding tissues, as well as prevention of chronic inflammation and lymphatic obstruction
Length of Hospital Stay	Baseline	Reduced by 3.7 days	This is driven by the earlier removal and recovery of drainage due to the effective sealing of lymphatic leakage.
* BCRL: Breast Cancer-Related Lymphedema.			
Clinical data for BCRL and Length of Stay are primarily derived from studies by 6, 10, 23.			
Reductions in hospital stay may be influenced by local drain management and discharge protocols.			

* BCRL: Breast Cancer-Related Lymphedema

**Table 4:** Illustrative Cost Domains Across the Continuum of Care in ALND.

Cost Domain	Primary Cost Driver	Conventional ALND	Advanced Energy Device-Assisted ALND	Total Sealing Technique (TST) Using LigaSure™ Exact	Notes
Inpatient hospitalization	Length of hospital stay (LOS)	Higher	Moderately reduced	Markedly reduced	LOS reduction reflects relative change within original study settings; absolute LOS varies by institution
Postoperative drainage management	Drain duration and volume	Prolonged	Shorter	Shortest	Earlier drain removal may not directly affect LOS in all systems but may influence recovery
Seroma management	Outpatient visits and aspirations	Frequent	Variable reduction	Substantially reduced	Reduced number of aspirations lowers direct and indirect outpatient costs
Device-related cost	Disposable instruments	Minimal	Higher	Higher	Incremental device cost should be interpreted in context of downstream savings
Long-term morbidity	BCRL-related care	Higher cumulative cost	Not evaluated	Substantially reduced	BCRL prevention has major long-term economic implications
Overall economic impact	Expected per-patient resource use	Higher	Moderately reduced	Lower (illustrative)	While the economic impact is exploratory and situation-dependent, TST has the potential to deliver significant cost savings.

The economic impact is presented as an illustrative model to reflect potential resource optimization; absolute costs may vary depending on regional healthcare systems and institutional discharge policies.

## Data Availability

The data presented in this study are available on request from the corresponding author.

## Abbreviations

The following abbreviations are used in this manuscript:

ALND	Axillary lymph node dissection
BCRL	Breast cancer-related lymphedema
SLNB	Sentinel lymph node biopsy
TST	Total sealing technique
CONV	Conventional electrocautery
ILR	Immediate lymphatic reconstruction
LGSED	LigaSure™ Exact Dissector
